# Congenital Heart Diseases and Periodontal Diseases—Is There a Link?

**DOI:** 10.3389/fcvm.2022.937480

**Published:** 2022-06-30

**Authors:** Roshan R. Rughwani, Priyanka K. Cholan, Dhayanand J. Victor

**Affiliations:** Department of Periodontics and Oral Implantology, SRM Dental College, Chennai, India

**Keywords:** periodontitis, congenital cardiac diseases, periodontal medicine, infective endocarditis, bacteremia, linking mechanism

## Abstract

An understanding in the field of periodontal medicine explains the fact that the oral cavity serves as a niche for numerous pathogenic microorganisms. When these microorganisms or their by-products disseminate to the various parts of the body, they are capable of triggering diseases characterized by an altered host immune-inflammatory response in the anatomically distinct organ. This mechanism is reported in the propagation of cardiovascular diseases with respect to periodontal medicine. Abundant amount of literature suggests an association between atherosclerotic cardiovascular disease and periodontal diseases. However, there is very less data available to highlight the association between periodontal disease and non-atherosclerotic cardiovascular disease, such as congenital anomalies of the heart. This review outlines the relationship between periodontal diseases and congenital heart diseases and also helps us understand whether the presence of periodontal disease can worsen the preexisting congenital cardiac disease.

## Introduction

The oral cavity gives asylum to commensal microflora, which is most commonly affected by prevalent human infectious diseases, such as dental caries and periodontitis (1). The theory of focal infection, which was proclaimed for more than a decade around nineteenth and twentieth centuries, states that focal sepsis, which are caused by oral microorganisms, is culpable of initiation and progression of many inflammatory diseases. This focal theory states that there is a casual link between most common oral diseases with systemic diseases, such as cardiovascular disease, diabetes, male and female reproductive disorders, osteoporosis, rheumatoid arthritis, neurodegenerative disorders, pulmonary diseases, and even cancers, namely, colorectal cancer and lung cancer. There is remarkable evidence that there is a presence of periodontal pathogens in artherosclerotic plaque of coronary health disease thus stating that periodontitis may be a risk factor for development of cardiovascular diseases (2, 3). However, the nature of this association needs to be further studied and a reasonable understanding needs to be derived to indoctrinate whether this association correlates periodontal disease with congenital cardiac diseases, which are caused due to genetic reasons.

## The Role of Periodontitis in Augmenting Immune-Inflammatory Pathways

In periodontitis, the pathogens in the biofilm, as well as their virulence factors dysregulate the normal symbiotic relationship between the host and the pathogen, leading to an exaggeration in the host immune response (4). The hazardous microenvironment created by this dysbiotic microflora triggers an immune response that is pronounced at the locus of the biofilm, leading to exponential damage of the periodontal tissues, which is the key triggering factor responsible for progression of gingivitis to periodontitis (5). Initially, phagocytes, such as the neutrophils and the macrophages, transmigrate to the site of bacterial insult, which produces a wide array of chemical mediators, such as the interleukins and prostaglandins, that not only sustain the process of inflammation locally but also aid in signaling of inflammatory cells to migrate to the site of periodontal destruction (6). These phagocytic cells express Toll-like receptors (TLRs) on their surface that are specialized to sense pathogen-associated molecular patterns (PAMPs) leading to a cascade of events through the MyD88-dependent pathway inducing the production of NFκB, that controls release of proinflammatory cytokines, such as interleukin (IL)-1β, tumor necrosis factor (TNF)-α, and IL-6 (7). This initial response to the biofilm initiates phagocytosis of the microorganisms and enables the elimination of the microbes that infringe on periodontal health.

The complement system gets activated synchronously, which enhances the susceptibility of these pathogens to the phagocytic action of neutrophils and macrophages. However, with the continuing maturation of the biofilm by pathobionts, there is a switch in the host to an adaptive immune response, wherein the bacterial antigen is sensed and processed by antigen presenting cells to the lymphocytes. This, in turn, leads to the differentiation and maturation of T-cells, B-cells, and the monocytes, which induce bone resorption and the dissolution of the periodontal ligament fibers through the coupling action of osteoclasts, and the matrix metalloproteinase (MMP)-mediated release of chemical mediators, such as RANKL, IL-1β, TNF-α, and IL-6, that promote osteoclastogenesis through various inflammatory signaling pathways (8). The persistent and virulent nature of the periodontal pathogens and their continually exaggerated elaboration of proinflammatory cytokines that disseminate into the systemic circulation serve as the bedrock for the initiation and progression of destructive inflammatory diseases in anatomically distinct organs that are away from the initiator of the disease—periodontal microbiome. This is basis to the science of periodontal medicine, which highlights the probable link between periodontal disease and systemic wellbeing, as well as the development of diseases, such as cardiovascular diseases, respiratory diseases, diabetes, rheumatoid arthritis, and reproductive disorders. This risk is further highlighted when the effects of periodontal diseases worsen the sufferings faced by patients with cardiovascular diseases.

## Clinico-Immunological Profile in Congenital Heart Diseases

Cardiovascular diseases encompass a wide range of disorders that arise out of atherosclerotic and non-atherosclerotic origins. Atherosclerosis is a leading cause of vascular disease worldwide, and its major clinical manifestations include ischemic heart diseases, stroke, and peripheral artery disease. In high-income countries, there have been dramatic declines in the incidence and mortality from ischemic heart disease and ischemic stroke since the middle of the twentieth century, which suggests that adequate awareness, research, and treatment protocols are available to address these issues (9). However, non-atherosclerotic cardiovascular diseases, such as congenital heart disease (CHD), encompass abnormalities in heart structures that occurs before birth, mainly due to genetic or chromosomal abnormalities (10).

Mitchell et al. defined CHDs as “a gross structural abnormality of the heart or intrathoracic great vessels that is actually or potentially of functional significance” (11). It occurs due to excessive alcohol consumption, judicious use of medication, and maternal viral infection during 1st trimester of pregnancy (12). With the advent of better medical facilities, the epidemiological figures denoting the mortality rate associated with CHDs seem to have improved, but the morbidity rate is still alarming. It is suggested that CHD affects 1 of every 100 live births (13). Although the occurrence of CHD may attribute to many causes, 15% of the known cases of CHD can be mapped to a known etiology, thereby making it imperative for us to understand the etiological role of periodontal disease in the augmentation of CHDs (14).

There are two common forms of CHDs, namely, cyanotic and acyanotic, which are based on the amount of deoxygenated hemoglobin present in blood (15, 16). However the most commonly occurring CHDs are ventricular septal defects, atrial septal defects, patent ductus arteriosus, pulmonary stenosis, coarction, and tetralogy of fallot (TOF), which account for almost 80% of all the congenital diseases that occur in humans (17).

Tetralogy of fallot, the most common congenital cardiac disease, comprises ventricular septal defects, aortic overriding, infundibular stenosis, and hypertrophy right ventricle. There are no precise symptoms for CHDs. However, shortness of breath, limited ability to do exercise, fatigue, and abnormal sounds of heart as heart murmurs are noticed (12).

These congenital anomalies may occur alone or together or as a manifestation of syndromes. The most common syndromes associated with congenital cardiac diseases are Down, Edwards, Digeorge, Hurler, Noonan, Treacher Collin, and Turner’s syndrome. These syndromes are associated with a multitude of effects that not only increase the morbidity but also play a role in augmenting the immune-inflammatory profile in these syndromes thereby having a direct effect on the pathogenesis of congenital cardiac diseases. It is reported that Down’s syndrome is associated with an increased TLR2 activity; however, there is a decrease in the activity of B- and T-lymphocytes and IgG production (18, 19). It has been suggested that elevated levels of IL-6 and TNF-α are seen in patients with Turner’s syndrome (20). Overall, patients suffering with stand-alone congenital cardiac diseases or as a manifestation of a syndrome are known to have weak immune responses characterized by decreased T-cell maturation and an increase in the suppressors of T-cell function, which eventually decreases the quality and quantity of B- and T-lymphocytes (21–23). An alteration in the immune response in CHDs can pose an array of effects on the systemic wellbeing of the individuals, as well as the periodontal health.

## Possible Association Between Periodontal Disease and Congenital Heart Disease

A preliminary report was published by Kaner et al. to evaluate oral findings in CHDs. In the TOF, the most noticeable abnormalities were seen in the tongue papillae, mucosal membranes, and gingivae. Similar but less pronounced structural alterations were observed in Eisenmenger’s tetralogy and in the transposition of the major vessels. The pulp canals in the maxillary incisors were observed to be significantly enlarged and funnel-shaped in patients with aortic coarctation. Dextrocardia, patent ductus arteriosus, and septal abnormalities all had no visible oral symptoms (24).

Due to the systemic effect of congenital cardiac diseases, the developing dentition also gets affected (25). Spivack discussed the dental implications of TOF after which Gedik et al. also showed an increase in dental caries, hypoplasia, and periodontal disease in the patients with TOF compared with controls (26, 27). When compared with gender- and age-matched healthy controls, nearly twice as many teeth in a sample of 60 children with severe congenital heart abnormalities showed symptoms of bacteria-induced gingivitis (28). This was supported in two recent studies, which indicated that children with CHDs have a higher gingival index (29, 30). Furthermore, more than two-thirds of the youngsters in one of these trials showed evidence of gingival and/or periodontal inflammation (30).

Various medications that are used to manage children with congenital cardiac disease found to have impact on oral health by altering saliva, plaque, mucosa, and gingiva. Also, the association between dental caries and digoxin has also been proved (31, 32). Similarly, beta-blockers and diuretics are known to induce a significant xerostomia and lichenoid reaction (33, 34). angiotensin-converting enzyme (ACE) inhibitors, such as captopril and enalapril, and calcium channel blockers, such as nifedipine, are known to cause gingival hyperplasia, while the use of oral anticoagulants may lead to bleeding gums (35, 36). The fact that children with congenital cardiac disease usually suffer with poor periodontal health compared with healthy children may be due to inadequate professional and personal dental care or due to an alteration in the immune response experienced in CHD; however, none of the studies explain the actual pathophysiological mechanism that links periodontal health and CHDs.

## Mechanisms Linking Periodontal Diseases and Congenital Heart Diseases

Pathogens present in the periodontal pockets are frequently present in the systemic circulation, which may get localized to anatomically defective sites of the cardiovascular system. A study analyzed the thrombus tissues isolated from defective aortic valves, mitral valve, and aortic aneurysmal wall specimens and reported that *Streptococcus mutans* was the most abundant bacteria followed by *Actinobacillus actinomycetemcomitans* (37). Oliveira et al. reported the presence of oral bacteria in heart valves by PCR analysis, which showed the most abundant oral microorganism to be *Streptococcus mutans* followed by periodontal pathogens, such as *Prevotella intermedia* and *Porphyromonas gingivalis* (38). The isolation of these microorganisms from diseased or defective cardiac tissues not only proves their presence in congenital heart defects but also serves as bedrock for the initiation of infective endocarditis in subjects with preexisting CHDs.

Recently, it is shown that bacteremia not only leads to the seeding of periodontal pathogens in the diseased or defective cardiovascular tissues but also causes the engagement of these bacteria or their byproducts in the liver that triggers the activation of inflammatory mediators, which forms the basis of an indirect mechanism associating periodontal diseases to congenital cardiovascular diseases (39). Periodontal disease is also known to elevate the level of several systemic biomarkers that cause endothelial dysfunction and dyslipidemia, such as the C-reactive protein (CRP), low-density lipoprotein (LDL), TNF-α, IL-6, and IL-1β (40). These findings were also recently supported by results from a study that elicited an elevation in the level of vascular inflammatory mediators after injecting rats with *P. gingivalis* LPS (41). Although CRP is a non-specific marker of inflammation, studies suggest that CRP is elevated in periodontal diseases, which further complicates the health status of the patient with CHD (42).

**FIGURE 1:**
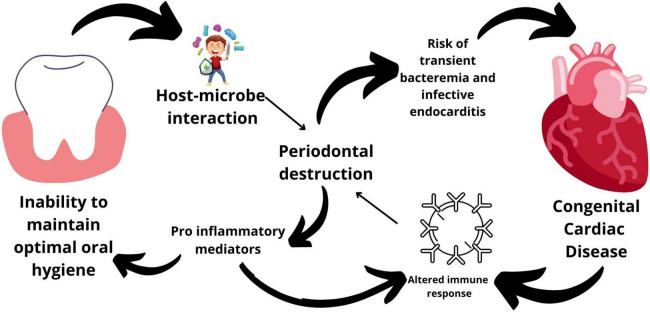
Mechanisms linking periodontal disease and congenital heart diseases.

Conversely, in both saliva and gingival crevicular fluid samples, the levels of IL-1β and PGE2 were considerably higher in patients with CHD compared with controls (43). Furthermore, greater levels of red complex bacterial counts were correlated with higher amounts of IL-1β in gingival crevicular fluid (43). PGE2 and IL-1β remained elevated in patients with CHD after correcting for gingivitis and plaque scores, indicating a systemic inflammatory component in both gingival crevicular fluid and saliva responsible for the maintenance of an inflammatory state in the periodontium of subjects with CHD (43). Hence, it can be understood that individuals with CHD have a greater systemic inflammation, which can be reflected in their oral fluids thereby linking periodontal and systemic inflammation (Figure 1).

## Systemic Effect of Periodontal Diseases and Bacterial Endocarditis in Congenital Cardiac Disease and Periodontitis Patients

A typical tooth with periodontitis can nurture 10^7^–10^8^ bacteria in the subgingival pocket. Periodontal pockets are lined with ulcerated epithelium, which paves way for direct bacterial contact with both the subadjacent connective tissue and inflammatory infiltrate (44). Thus, high number of bacterial load, which are found to be associated with dental plaque and gingivitis, leads patients with cardiac disease into the risk of developing bacterial endocarditis (45). It is reasonable to infer that adult patients with CHD and poor oral hygiene are more likely to acquire periodontitis than healthy people. As a result, people with CHD who have periodontitis may be at an increased risk of infective endocarditis (46).

Periodontal pathogens cause bacteremia not only by directly invading the periodontal tissue, but also by paving a way for direct bacterial translocation when the epithelial integrity is lost within the periodontal pocket. This transient bacteremia is increased in patients with periodontal issues even while brushing the teeth or chewing the food, thereby explaining the cause for isolation of periodontal pathogens from samples obtained from the diseased cardiovascular tissues (47–50).

Several investigators have found periodontal pathogens, such as *P. gingivalis* and *A. actinomycetocomitants*, in the specimens of cardiovascular patients. *P. gingivalis* is found to play a role in aggregation of platelets, thereby playing a major role in homeostasis and thrombosis. Also, *P. gingivalis* is found to invade the aortic valve and endothelial cells *via* their fimbriae (51–53).

Patients with congenital cardiac disease are more susceptible to bacterial endocarditis due to poor oral hygiene, which is caused mainly by bacterial invasion and its consequences that alter the blood rheology. Since gingival and periodontal diseases are the prime sources of bacterial invasion, understanding the association of poor oral health and preexisting CHD becomes indisputable. It is suggested that *streptococci* and *staphylococci* collectively account for 80% of the infective endocarditis cases (54). In some cases, even the diagnostic probing and subgingival scaling of periodontal pocket may cause soft-tissue damage accounting for 15–25% of transient bacteremia, which was evidenced by the findings of Hunter KM, which increases 1,000 times more after dental procedures (55). According to the American Association for Periodontology, 1 out of 4 infective endocarditis is caused by periodontal germs, such as *Eikenella corrodens*, a bacteria, which is highly relevant in juvenile periodontitis (56).

Most of the congenital anomalies pose an additional burden of the overall metabolism of the body, and the challenges that are faced by the patient often include fatigue, dyspnea on exertion, and arrhythmias in addition to the fatal risk of developing cyanosis and infective endocarditis. Children with congenital anomalies of the heart are mostly chronically sick, and expecting them to maintain good oral hygiene is not as easy as it can be expected from apparently healthy children. Such children visit hospitals on multiple occasions for checkups and treatments where in the emphasis on dental care is minimal, owing to the conduction of extensive cardiovascular therapies, thereby neglecting oral hygiene of such subjects. Hence, oral hygiene maintenance and enforcement become important in comprehensively managing such patients. Most children might also have undergone cardiac surgery prior to meeting a dentist in their life. Such children might be apprehensive or even anxious, and anxiety control becomes an important pillar based on which further dental treatment can be carried out in such children. However, the following steps must be taken to deliver optimal oral care to patients with CHDs:

1. Documenting a comprehensive medical and social history.
2. Specific inquest about the original defect, current medications, and previous treatment should be noted.
3. History regarding their oral care and dietary practices should be elicited.
4. After complete history and examination, an explicit diagnosis can be made and treatment plan can be formulated.
5. Collaboration with cardiologist or family medical practitioner is advised before providing active treatment. This can be procured in the form of letter.
6. It is important to provide preventive dentistry treatment, such as dietary advice, fluoride therapy, and oral hygiene maintenance, to all the patients with congenital cardiac disease.
7. Children should be advised to avoid vigorous brushing.
8. Appointments should be given in the morning with shorter duration, also with intervals of 2–4 weeks (25).
9. Before commencing dental procedure, children should be advised to rinse with 0.2% *Chlorhexidine* mouthwash.
10. Antibiotic prophylaxis should be given before dental procedures, such as extractions, dental prophylaxis, and surgeries, to place or remove orthodontic bands, local anesthesia injection, periodontal procedures, tooth replantation, and any bleeding procedure (57).
11. Pulp therapy should be carried out before extraction especially in primary dentition.
12. Stainless steel crown is often indicated to direct intracoronal restoration, especially for deciduous teeth. The reason to prefer stainless steel crown is it offers minimal technique sensitivity and often full coronal coverage (58, 59).
13. Chronic periodontitis without any signs or symptoms of infection should be monitored, and scaling and oral hygiene have to be provided.
14. Teeth with advanced periodontal disease need removal prior to surgery.
15. Extraction, periodontal, and other surgical treatment should be planned carefully due to the presence of preexisting coagulopathies.
16. Patients those who are warfarinized should undergo extraction with international normalized ratio (INR) up to 4. An INR should be obtained within 24 h of proposed surgery (25).
17. Postoperative bleeding can be controlled by placing hemostatic gauze, sponges, and sutures (15, 60).

Patients with congenital cardiac disease need special care in dentistry as they are more susceptible to infective endocarditis due to periodontitis. It is mandatory that adults, who are having congenital cardiac diseases, need to be periodontally treated regularly, considering the abovementioned points to avoid unnecessary systemic risks of bacterial invasion.

## Conclusion

These studies suggest that the presence of poor oral hygiene and increased chances of gingival inflammation in patients with congenital anomalies of the heart is merely casual or due to general weakness that prevents the patient from maintaining optimal oral hygiene, thereby emphasizing the importance of administrating prophylactic antibiotics before dental procedures in order to prevent bacterial endocarditis. There exists an ambiguity in the preexisting data that explain the effect of altered immune responses in CHDs as studies have reported a decrease in the function of the B- and T-cell subsets, which may affect the periodontium. The altered immune-inflammatory mechanisms may bridge the lacunae in establishing the association between periodontal diseases and congenital cardiac diseases. However, long-term interventional studies are needed to understand the nature of association between both the diseases.

## Author Contributions

RR generated the manuscript and the graphical image. PC assisted RR to correct the manuscript. DV assisted in proofreading the manuscript and correcting it along with collection of relevant data and articles. All authors contributed to the article and approved the submitted version.

## Conflict of Interest

The authors declare that the research was conducted in the absence of any commercial or financial relationships that could be construed as a potential conflict of interest.

## Publisher’s Note

All claims expressed in this article are solely those of the authors and do not necessarily represent those of their affiliated organizations, or those of the publisher, the editors and the reviewers. Any product that may be evaluated in this article, or claim that may be made by its manufacturer, is not guaranteed or endorsed by the publisher.

## References

[1] Kankara VR Reddy NR. Congenital heart disease and its journey from dental plaque to arterial plaque. *J Int Clin Dent Res Organ.* (2016) 8:137.

[2] Li X Kolltveit KM Tronstad L Olsen I. Systemic diseases caused by oral infection. *Clin Microbiol Rev.* (2000) 13:547–58. 10.1128/CMR.13.4.547 11023956 PMC88948

[3] D’Aiuto F Parkar M Tonetti MS. Acute effects of periodontal therapy on bio-markers of vascular health. *J Clin Periodontol.* (2007) 34:124–9. 10.1111/j.1600-051X.2006.01037.x 17214734

[4] D’Aiuto F Graziani F Tetè S Gabriele M Tonetti MS. Periodontitis: from local infection to systemic diseases. *Int J Immunopathol Pharmacol.* (2005) 18(Suppl. 3):1–11. 16848982

[5] Sudhakara P Gupta A Bhardwaj A Wilson A. Oral dysbiotic communities and their implications in systemic diseases. *Dent J.* (2018) 6:1–14. 29659479 10.3390/dj6020010 PMC6023521

[6] Metcalfe S Anselmi N Escobar A Visser MB Kay JG. Innate phagocyte polarization in the oral cavity. *Front Immunol.* (2022) 12:768479. 10.3389/fimmu.2021.768479 35069541 PMC8770816

[7] Song B Zhang YL Chen LJ Zhou T Huang WK Zhou X The role of Toll-like receptors in periodontitis. *Oral Dis.* (2017) 23: 168–80. 26923115 10.1111/odi.12468

[8] Balaji S Cholan PK Victor DJ Appukuttan D. Development and activation of T cell subsets – An overview from a periodontal perspective. *Eur J Mol Clin Med.* (2020) 7:4489–501.

[9] Herrington W Lacey B Sherliker P Armitage J Lewington S. Epidemiology of atherosclerosis and the potential to reduce the global burden of atherothrombotic disease. *Circ Res.* (2016) 118:535–46. 10.1161/CIRCRESAHA.115.307611 26892956

[10] Fahed AC Gelb BD Seidman JG Seidman CE. Genetics of congenital heart disease: the glass half empty. *Circ Res.* (2013) 112:707–20. 10.1161/CIRCRESAHA.112.300853 23410880 PMC3827691

[11] Mitchell SC Korones SB Berendes HW. Congenital heart disease in 56,109 births. Incidence and natural history. *Circulation.* (1971) 43:323–32. 10.1161/01.cir.43.3.323 5102136

[12] Sun R Liu M Lu L Zheng Y Zhang P. Congenital heart disease: causes, diagnosis, symptoms, and treatments. *Cell Biochem Biophys.* (2015) 72:857–60. 10.1007/s12013-015-0551-6 25638345

[13] Gruber PJ. Cardiac development: new concepts. *Clin Perinatol.* (2005) 32:845–55. 10.1016/j.clp.2005.09.003 16325665

[14] Wu W He J Shao X. Incidence and mortality trend of congenital heart disease at the global, regional, and national level, 1990-2017. *Medicine (Baltimore).* (2020) 99:e20593. 10.1097/MD.0000000000020593 32502030 PMC7306355

[15] Hallett KB Radford DJ Seow WK. Oral health of children with congenital cardiac diseases: a controlled study. *Pediatr Dent.* (1992) 14:224–30. 1303520

[16] Weddell JA Sanders BJ Jones JE. Chapter 25: Dental problems of children with special health care needs. 10th ed. In: Dean JA Avery DR McDonald RE editors. *McDonald and Avery’s Dentistry for the Child and Adolescent.* Amsterdam: Elsevier Inc. (2016). p. 513–39.

[17] Gatzoulis MA Swan L Therrien J Pantely GA. Epidemiology of congenital heart disease. In: Gatzoulis MA Swan L Therrien J Pantely GA editors. *Adult Congenital Heart Disease: A Practical Guide.* Hoboken, NJ: John Wiley & Sons, Ltd (2005). p. 3–7.

[18] Ram G Chinen J. Infections and immunodeficiency in Down syndrome. *Clin Exp Immunol.* (2011) 164:9–16. 10.1111/j.1365-2249.2011.04335.x 21352207 PMC3074212

[19] Huggard D Koay WJ Kelly L Mcgrane F Ryan E Lagan N Altered toll-like receptor signalling in children with down syndrome. *Mediators Inflamm.* (2019) 2019:4068734. 10.1155/2019/4068734 31611734 PMC6757445

[20] Su MA Stenerson M Liu W Putnam A Conte F Bluestone JA The role of X-linked FOXP3 in the autoimmune susceptibility of Turner Syndrome patients. *Clin Immunol.* (2009) 131:139–44. 10.1016/j.clim.2008.11.007 19150256 PMC2949956

[21] Khalil A Trehan R Tiwari A Malik R Arora R. Immunological profile in congenital heart disease. *Indian Pediatr.* (1994) 31:295–300. 7896364

[22] Parikh S Bharucha B Kamdar S Kshirsagar N. Polymorphonuclear leukocyte functions in children with cyanotic and acyanotic congenital heart disease. *Indian Pediatr.* (1993) 30:883–90. 8132280

[23] Rhoden DK Leatherbury L Helman S Gaffney M Strong WB Guill MF. Abnormalities in lymphocyte populations in infants with neural crest cardiovascular defects. *Pediatr Cardiol.* (1996) 17:143–9. 10.1007/BF02505203 8662026

[24] Kaner A Losch PK Green H. Oral manifestations of congenital heart disease. *J Pediatr.* (1946) 29:269–74. 10.1016/s0022-3476(46)80140-9 21002141

[25] FitzGerald K Fleming P Franklin O. Dental health and management for children with congenital heart disease. *Prim Dent Care.* (2010) 17:21–5. 10.1308/135576110790307690 20067687

[26] Spivack E. Tetralogy of Fallot: an overview, case report, and discussion of dental implications. *Spec Care Dent.* (2001) 21:172–5. 10.1111/j.1754-4505.2001.tb00250.x 11803640

[27] Gedik S Gedik R Gedik T. Tetralogy of fallot: report of 30 cases and dental considerations with review of literature. *WIMJ Open.* (2015) 2:102–5.

[28] Franco E Saunders CP Roberts GJ Suwanprasit A. Dental disease, caries related microflora and salivary IgA of children with severe congenital cardiac disease: an epidemiological and oral microbial survey. *Pediatr Dent.* (1996) 18:228–35. 8784915

[29] Ali HM Berggreen E Nguyen D Ali RW Van Dyke TE Hasturk H Dental plaque microbial profiles of children from Khartoum, Sudan, with congenital heart defects. *J Oral Microbiol.* (2017) 9:1281556. 10.1080/20002297.2017.1281556 28326155 PMC5328311

[30] Pourmoghaddas Z Meskin M Sabri M Norousali Tehrani MH Najafi T. Dental caries and gingival evaluation in children with congenital heart disease. *Int J Prev Med.* (2018) 9:52. 10.4103/ijpvm.IJPVM_401_15 30034670 PMC6028990

[31] Stecksén-Blicks C Rydberg A Nyman L Asplund S Svanberg C. Dental caries experience in children with congenital heart disease: a case-control study. *Int J Paediatr Dent.* (2004) 14:94–100. 10.1111/j.1365-263x.2004.00531.x 15005697

[32] Moursi AM Fernandez JB Daronch M Zee L Jones CL. Nutrition and oral health considerations in children with special health care needs: implications for oral health care providers. *Pediatr Dent.* (2010) 32:333–42. 20836954

[33] Smith RG Burtner AP. Oral side-effects of the most frequently prescribed drugs. *Spec Care Dent Off Publ Am Assoc Hosp Dent Acad Dent Handicap Am Soc Geriatr Dent.* (1994) 14:96–102. 10.1111/j.1754-4505.1994.tb01112.x 7871475

[34] Rosén L Stecksén-Blicks C. Experience of dental care for children with congenital heart disease among Swedish dentists. *Swed Dent J.* (2007) 31:85–90. 17695053

[35] Pradhan S Mishra P. Gingival enlargement in antihypertensive medication. *JNMA J Nepal Med Assoc.* (2009) 48:149–52. 20387357

[36] Seymour RA. Calcium channel blockers and gingival overgrowth. *Br Dent J.* (1991) 170:376–9. 2064860 10.1038/sj.bdj.4807564

[37] Nakano K Inaba H Nomura R Nemoto H Takeda M Yoshioka H Detection of cariogenic Streptococcus mutans in extirpated heart valve and atheromatous plaque specimens. *J Clin Microbiol.* (2006) 44:3313–7. 10.1128/JCM.00377-06 16954266 PMC1594668

[38] Oliveira FAF Forte CPF Silva PGB Lopes CB Montenegro RC Dos Santos ÂKCR Molecular analysis of oral bacteria in heart valve of patients with cardiovascular disease by real-time polymerase chain reaction. *Medicine (Baltimore).* (2015) 94:e2067. 10.1097/MD.0000000000002067 26632711 PMC5058980

[39] Schenkein HA Papapanou PN Genco R Sanz M. Mechanisms underlying the association between periodontitis and atherosclerotic disease. *Periodontol 2000.* (2020) 83:90–106. 10.1111/prd.12304 32385879

[40] Joshipura K. The relationship between oral conditions and ischemic stroke and peripheral vascular disease. *J Am Dent Assoc.* (2002) 133:23S–30S. 10.14219/jada.archive.2002.0373 12085721

[41] Leira Y Iglesias-Rey R Gómez-Lado N Aguiar P Sobrino T D’Aiuto F Periodontitis and vascular inflammatory biomarkers: an experimental *in vivo* study in rats. *Odontology.* (2020) 108:202–12. 10.1007/s10266-019-00461-3 31583485 PMC7066291

[42] Bokhari SAH Khan AA Butt AK Azhar M Hanif M Izhar M Non-surgical periodontal therapy reduces coronary heart disease risk markers: a randomized controlled trial. *J Clin Periodontol.* (2012) 39:1065–74. 10.1111/j.1600-051X.2012.01942.x 22966824

[43] Mohamed H Manal A Salwa M Osama S Elshazali H Whahab R Inflammatory mediators in saliva and gingival fluid of children with congenital heart defect. *Oral Dis.* (2020) 26:1053–61. 10.1111/odi.13313 32100914

[44] Izumi Y Nagasawa T Umeda M Kobayashi H Takeuchi Y Yashiro R Periodontitis and cardiovascular diseases : the link and relevant mechanisms. *Jpn Dent Sci Rev.* (2009) 45:98–108.

[45] Steelman R Einzig S Balian A Thomas J Rosen D Gustafson R Increased susceptibility to gingival colonization by specific HACEK microbes in children with congenital heart disease. *J Clin Pediatr Dent.* (2000) 25:91–4. 10.17796/jcpd.25.1.kv5218kw3ql67r67 11314361

[46] Carinci F Martinelli M Contaldo M Santoro R Pezzetti F Lauritano D Focus on periodontal disease and development of endocarditis. *J Biol Regul Homeost Agents.* (2018) 32:143–7. 29460534

[47] Dhadse P Gattani D Mishra R. The link between periodontal disease and cardiovascular disease : how far we have come in last two decades ? *J Indian Soc Periodontol.* (2010) 14:148–54. 10.4103/0972-124X.75908 21760667 PMC3100856

[48] Armitage GC. Periodontal infections and cardiovascular disease–how strong is the association? *Oral Dis.* (2000) 6:335–50. 10.1111/j.1601-0825.2000.tb00126.x 11355266

[49] Beck J Garcia R Heiss G Vokonas PS Offenbacher S. Periodontal disease and cardiovascular disease. *J Periodontol.* (1996) 67:1123–37. 10.1902/jop.1996.67.10s.1123 29539797

[50] Folwaczny M Bauer F Grünberg C. Significance of oral health in adult patients with congenital heart disease. *Cardiovasc Diagn Ther.* (2019) 9(Suppl. 2):S377–87. 10.21037/cdt.2018.09.17 31737544 PMC6837931

[51] Chiu B. Multiple infections in carotid atherosclerotic plaques. *Am Heart J.* (1999) 138:4–6. 10.1016/s0002-8703(99)70294-2 10539867

[52] Haraszthy VI Zambon JJ Trevisan M Zeid M Genco RJ. Identification of Periodontal Pathogens in Atheromatous Plaques. *J Periodontol.* (2000) 71:1554–60. 10.1902/jop.2000.71.10.1554 11063387

[53] Deshpande RG Khan MB Genco CA. Invasion of aortic and heart endothelial cells by Porphyromonas gingivalis. *Infect Immun.* (1998) 66:5337–43. 10.1128/IAI.66.11.5337-5343.1998 9784541 PMC108667

[54] Holland TL Baddour LM Bayer AS Hoen B Miro JM Fowler VGJ. Infective endocarditis. *Nat Rev Dis Prim.* (2016) 2:16059. 10.1038/nrdp.2016.59 PMC5240923 27582414

[55] Hunter KM Holborow DW Kardos TB Lee-Knight CT Ferguson MM. Bacteraemia and tissue damage resulting from air polishing. *Br Dent J.* (1989) 167:275–8. 10.1038/sj.bdj.4806998 2590584

[56] Suda R Lai C Yang H Hasegawa K. Eikenella corrodens in subgingival plaque: relationship to age and periodontal condition. *J Periodontol.* (2002) 73:886–91. 12211498 10.1902/jop.2002.73.8.886

[57] Hughes S Balmer R Moffat M Willcoxson F. The dental management of children with congenital heart disease following the publication of Paediatric Congenital Heart Disease Standards and Specifications. *Br Dent J.* (2019) 226:447–52. 10.1038/s41415-019-0094-0 30903073

[58] Seale NS. The use of stainless steel crowns. *Pediatr Dent.* (2002) 24:501–5. 12412965

[59] Kindelan SA Day P Nichol R Willmott N Fayle SA. UK National Clinical Guidelines in Paediatric Dentistry: stainless steel preformed crowns for primary molars. *Int J Paediatr Dent.* (2008) 18(Suppl. 1):20–8. 18808544 10.1111/j.1365-263X.2008.00935.x

[60] Balmer R Bu’Lock FA. The experiences with oral health and dental prevention of children with congenital heart disease. *Cardiol Young.* (2003) 13:439–43. 14694938 10.1017/s1047951103000921

